# Supplementary material for: Has the COVID-19 Pandemic Affected Cyberbullying and Cybervictimization Prevalence among Children and Adolescents? A Systematic Review
Source: Int J Environ Res Public Health. 2023 May 15;20(10):5825. doi: 10.3390/ijerph20105825 (PMC10218135; doi:10.3390/ijerph20105825)
Supplement: Supplementary file 1 [file ijerph-20-05825-s001.zip › ijerph-2254428-supplementary.pdf]

Table S1: *Quality assessment results based on Critical Appraisal tools in JBI Systematic Reviews for prevalence studies*

Study	1	2	3	4	5	6	7	8	9
Choi, Shin, and Lee [91]<br>South Korea	Yes	No	Yes	Yes	Yes	No	Yes	Yes	Yes
Eroglu et al. [96]<br>Turkey	Yes	Yes	Yes	Yes	Yes	Yes	No	Yes	Unclear
Mohd Fadhli et al. [94]<br>Malaysia	Yes	Yes	Yes	Yes	No	Yes	No	Yes	Unclear
Repo et al. [60]<br>Finland	Yes	No	Yes	Yes	Yes	No	No	Yes	Unclear
Rodriguez-Rivas et al. [102]<br>Chile	Yes	No	No	Yes	Yes	Yes	Yes	Yes	Yes
Schunk et al. [99]<br>Study 1<br>Germany	Yes	No	No	Yes	Yes	Yes	Yes	Yes	Yes
Thai et al. [97]<br>Vietnam	Yes	Yes	Yes	Yes	No	Yes	Unclear	Yes	Yes
Thumronglaohapun et al. [95]<br>Thailand	Yes	Yes	Yes	Yes	No	Yes	No	Yes	Yes
Trompeter et al. [100]<br>Australia	No	Unclear	No	Yes	No	Yes	No	Yes	Yes
Wiguna et al. [93]<br>Indonesia	Yes	No	Yes	Yes	No	No	Yes	Yes	Yes
Xiang et al. [89]<br>China	Yes	No	Yes	Yes	Yes	Yes	Yes	Yes	Yes
Zhao et al. [90]<br>China	Yes	No	Yes	Yes	No	Yes	Yes	Yes	Yes
Han et al. [52]<br>China	Yes	Yes	Yes	Yes	No	Yes	Yes	Yes	Yes
Shin and Choi [92]<br>South Korea	Yes	No	Yes	Yes	Yes	No	Yes	Yes	Yes
Vaillancourt et al. [101]<br>Canada	Yes	Yes	Yes	Yes	Yes	Yes	Yes	Yes	Yes
Vejmelka & Matković [98]<br>Croatia	Yes	Yes	Yes	No	Yes	Yes	Unclear	Yes	Yes

Table S2. *Description of studies excluded from the systematic review*

Study	Description
Paulus et al. [64]	The study investigated Problematic Internet Use. Cyberbullying and Cybervictimization were not measured<br>→ *No CB/CV outcomes*
Schacter et al.[65]	The study investigated participants' experiences of peer victimization without distinguish the context of such peer victimization behaviours<br>→ *No CB/CV outcomes*
Arnon et al.[66]	The study analysed longitudinal data collected from July 2018 and January 2021. Despite this, 72.0% of participants were surveyed before the COVID-19 pandemic.<br>→ *Wrong period of data collection*
González-Cabrera et al. [67]	Data collection was carried out from December 2017 to February 2019<br>→ *Wrong period of data collection*
Iorga et al. [68](	Data were collected between May and June 2022<br>→ *Wrong period of data collection*
Martínez-Martínez et al.[69]	Data collection took place pre COVID-19 during the second quarter of 2017 and 2018<br>→ *Wrong period of data collection*
Maurya et al.[70]	Longitudinal study referring to two waves of data collection. The first wave was in 2015–2016, with a follow-up after 3 years in 2018–2019<br>→ *Wrong period of data collection*
Sampasa-Kanyinga et al.[71]	The study included 7.229 students aged 11–20 years. Data were collected from November 2018 to June 2019<br>→ *Wrong period of data collection*
Sampasa-Kanyinga et al.[72]	The study included 6.834 adolescents aged 11-20 years. Data were collected from November 2018 to June 2019<br>→ *Wrong period of data collection*
Shah et al.[73]	Longitudinal study reporting data collected between 2014 and 2017<br>→ *Wrong period of data collection*
Shin and Kim [74]	Data were collected between 2010 and 2016<br>→ *Wrong period of data collection*
Trbojević & Šikuten [75]	The study involved 269 elementary school students in Croatia. Data were collected between December 2019 to January 2020, before the beginning of the pandemic and restriction measures<br>→ *Wrong period of data collection*
Barlett et al.[51]	The study involved 181 adult US participants reporting an average age of 37.21 (SD = 10.84) years<br>→ *Wrong population (age > 18)*
Barlett et al. [76](2021)	The study involved 194 US participants an average age 37.15 (SD = 10.33) years<br>→ *Wrong population (age > 18)*
Ch'ng, et al.[77] (2021)	Participants' age was > 18 years<br>→ *Wrong population (age > 18)*
Kee et al. [78]	The sample consisted of 200 Malaysian university students<br>→ *Wrong population (>18 age)*
Gajda et al. [79]	The sample involved 251 adults (M_{age} = 28.54, SD_{age} = 9.10; age range = 18–60)<br>→ *Wrong population (age > 18)*
Pomytkina et al. [80]	The study involved participants aged between 18 and 22 years<br>→ *Wrong population (age > 18)*
Varela et al.[81]	The sample was aged between 15 and 29 years<br>→ *Wrong population (age > 18)*

Shannen et al.[82]	Data collection period is not clear. Furthermore, the sample consists of two age categories from 12 to 15 age and > 18 age, CB and the CV were measured on the total sample<br>→ *Wrong population (age > 18) and no reference to data collection period*
Tintori et al. [83]	Data were collected during 2019 involving participants aged between 13 and 22 years<br>→ *Wrong population (age > 18) and period of data collection*
Feijóo et al. [84]	3.188 adolescents aged 12-17 years. No information about the data collection period were available<br>→ *No reference to the pandemic; no reference to the period of data collection*
Ibrahim and Vi [85]	992 secondary school adolescents aged from 15 to 17 years. No information about the data collection period were available.<br>→ *No reference to the pandemic; no reference to the period of data collection*
Yirci et al. [86]	This cross-sectional study involved 311 adolescent students from 9th to 12th grades. No information about the data collection period were available.<br>→ *No reference to the pandemic; no reference to the period of data collection*
Floros, & Mylona [87]	Systematic review on cyberbullying and Internet Use Disorder (IUD)<br>→ *Systematic review*
Zakuan and Saian [88]	Systematic review of 19 studies referring to cybervictimization and its psychological effect during COVID 19 pandemic.<br>→ *Systematic review*
